# Analysis of Energy Consumption in the Cutting Zone During Turning Bearing Steel 16MnCr5

**DOI:** 10.3390/ma18215059

**Published:** 2025-11-06

**Authors:** Anna Mičietová, Mário Drbúl, Mária Čilliková, Miroslav Neslušan

**Affiliations:** Faculty of Mechanical Engineering, University of Žilina, Univerzitná 1, 010 26 Žilina, Slovakia; anna.micietova@fstroj.uniza.sk (A.M.); mario.drbul@fstroj.uniza.sk (M.D.); maria.cillikova@fstroj.uniza.sk (M.Č.)

**Keywords:** turning, chip former, energy consumption, cutting force

## Abstract

This paper deals with the consumption of energy during the turning of low-alloyed steel 16MnCr5. The study employs the earlier reported methodology for the decomposition of energy in cutting during turning. The energy for chip formation, as well as the energy consumed in the interface between the tool flank and produced surfaces, can be singled out. The paper investigates the turning process as a function of the cutting conditions as well as the variable cutting edge geometry. It was found that the integration of a chip former valuably contributes to the lower chip ratios, as well as the more favourable shape of chips. The lower energy consumed in the tool flank region for the tool with the integrated chip former results in lower normal and shear forces despite the higher cutting edge radius. However, the differences in the surface strain accumulation expressed in terms of the dislocation density and residual stress depth profiles are only subtle.

## 1. Introduction

**Figure 1:**
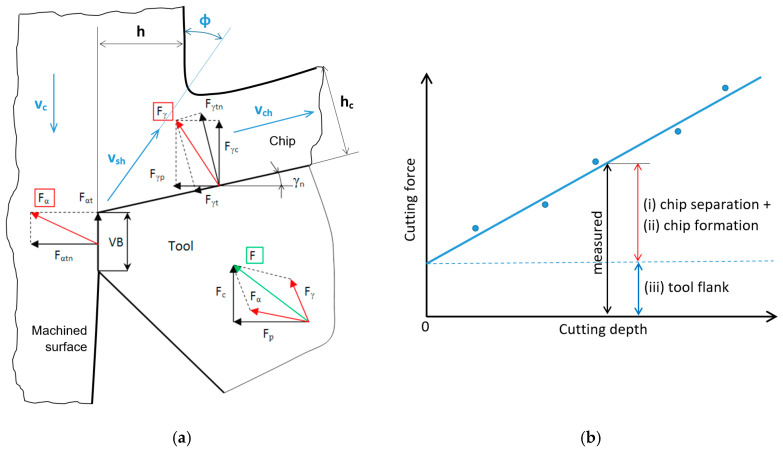
Decomposition of energy during turning. (**a**) Brief illustration of cutting force component directions; (**b**) separation of the energy consumed in the tool rake and flank regions.

The turning process consumes energy, especially for plastic deformation in the cutting zone, whereas the energy for the elastic regime and/or friction is less demanding. Plastic deformation in the cutting zone can be separated into the energy required for chip separation and formation, and the energy necessary for the formation of the new surface. Three basic regions have to be considered with respect to the cutting zone during turning, and are linked with the energy consumption, those being (i) the energy necessary for dislocation shearing following the angle of primary plastic deformation, which is usually predominating (along *v_sh_*); (ii) the friction in the cutting insert rake and chip interface (along *v_ch_*); and finally (iii) the friction in the insert flank and new formed surface region (along *v_c_*), as seen in Figure 1a. The first two components can be separated from the third component by using samples with a variable cutting depth [1] or by the measurement of cutting forces as a function of the cutting depth, as seen in Figure 1b (Equations (1) and (2), when *F_αtn_* and *F_αt_* are obtained from Figure 1b; *F_p_* and *F_c_* are measured). A certain idea about the energy consumed for chip separation can be obtained from the measurement of chip thickness and the corresponding chip ratio coefficient *K*, as discussed later [2,3].

(1) $${\bar{F}}_{\gamma p}={\bar{F}}_{p}-{\bar{F}}_{\alpha tn}\;\;\;(N)$$

(2) $${\bar{F}}_{\gamma c}={\bar{F}}_{c}-{\bar{F}}_{\alpha t\;}\;\;(N)$$

In contrast to the chip formation (which is linked with the dislocation motion along *v_sh_*), chip formation is linked with the further plastic deformation of the separated chip through the use of a suitable chip former. A chip former is employed in order to produce an acceptable chip shape, especially when the automated chip disposal from the cutting region is addressed. For this reason, the chip rake usually has quite a complicated geometry, which guarantees that chips of acceptable shape would be produced within a certain range of cutting conditions (especially feeds and cutting depths—well known as diagrams of chip formation). Two basic types can be used in real industrial applications, those being obstructive and groove ones. Chip formers are produced in a diversity of sizes and shapes for different applications with respect to the cutting conditions, technological processes, machined materials, etc. It is necessary to note that the application of a chip former also takes a certain role in chip separation, since the employment of a chip former alters the rake angle. These aspects take a significant role in chip thickness and its linked parameters, such as the angle of primary plastic deformation, chip speed, shear speed, etc. [3,4]. For these reasons, the chip separation and the chip formation are mutually connected processes that depend on each other.

Plastic deformation in the region of the tool flank is linked with the presence of flank wear and/or cutting edge wear, as illustrated in Figure 1a, or/and the presence of a certain cutting edge radius, *r_n_*, which cannot be avoided. On the contrary, a certain cutting edge radius is recommended in order to make the cutting edge more stable against microcracking or/and make tool wear lower [5,6]. Furthermore, the mechanical and linked thermal loads in this region are linked with the surface state expressed in such terms as the surface hardening measured by micro- or nano-indentation techniques [7,8], dislocation density calculated from the XRD patterns of KAM images [9,10], residual stress state [11,12], phase alterations [13,14], etc.

Plastic deformation during machining is a very complicated and complex process under elevated temperatures and extreme strain rates, when the distribution of temperature and stress fields is very heterogeneous. For this reason, strong stress and temperature gradients should be considered in order to avoid an oversimplification of the problems analysed. This study provides quite complex insights into the turning process in order to distinguish among the different regions mentioned earlier and discusses the influence of aspects of chip separation and formation, as well as the surface state.

## 2. Materials and Methods

**Figure 2:**
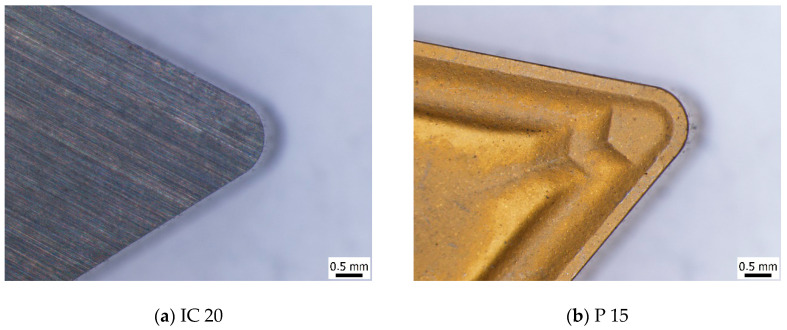
Cutting inserts employed for turning process. (**a**) IC 20; (**b**) P 15.

**Figure 3:**
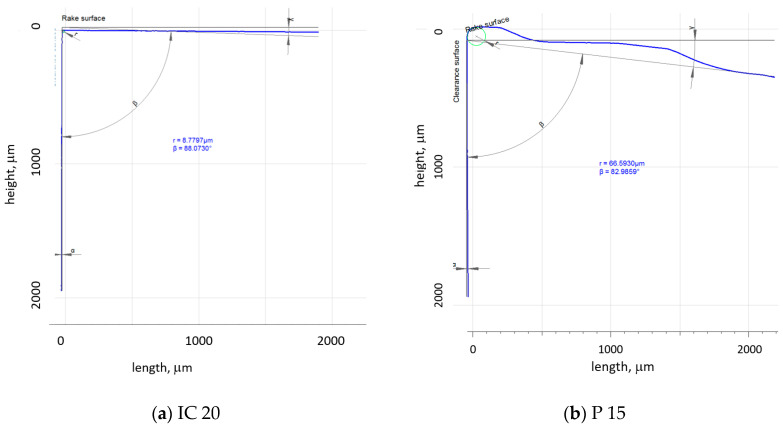
Cutting inserts geometry. (**a**) IC 20; (**b**) P 15.

Experiments were carried out on the annealed steel 16MnCr5 (of hardness 30.5 ± 2.2 *HV1*) in the form of a rod of diameter 75 mm. The rod initial runout of the as-received body and its corroder surface were removed during the preliminary phase of the turning process. The chemical composition of this steel is indicated in Table 1. The turning process was carried out on the lathe SUI 40 by using the tool holder NAREX PN 0524.0 25 × 25 and two different TNMA 220408 inserts: IC 20 (without a chip former or coating) and P15, with the chip former as well as TiN coating; see Figure 2. Further cutting conditions: dry turning, *f* = 0.09 ÷ 0.225 mm, *a_p_* = 0.25 ÷ 1 mm, *v_c_* = 50 ÷ 150 m.min^−1^. The insert’s cutting edge profiles are depicted in Figure 3.

The cutting edge geometry was measured by the Alicona 5 device (IF-Edge MasterModule, Bruker, Dallas, TX, USA). All extracted parameters were obtained by averaging 20 measurements. Table 2 compares the employed inserts with respect to their geometry. The components of the cutting force, *F_c_* and *F_p_*, were measured using the dynamometer Kistler 9441 (Kistler Instrumente GmbH, Sindelfingen, Germany) in the DasyLab software (measX GmbH & Co. KG, Moenchengladbach, Germany) (sampling frequency 2 kHz, low-pass filter 20 Hz). Chip thickness was measured by the light microscope Zeiss AxioCam MRc5 (ZEISS Group, Wetzlar, Germany) and Olympus SZx16 in the Quick Photo Industrial 3.0 software (Olympus Europa SE & Co. KG, Hamburg, Germany). The chips were not straightened before the measurements of their thickness. The chip thickness was measured in 20 positions (the consecutive measurements along the chip length spaced 5 mm from each to other). The measured positions were far away from the chip’s beginning as well as its end (about 30 mm). Calibration of the optics was carried out before the measurement by the measurement of a calibration sample of known thickness (0.52 mm).

Residual stress depth profiles were measured using the X-ray diffraction (XRD) technique. The XRD patterns were also employed for the assessment of dislocation density, having information about the *FWHM* (full width at half maximum) of the diffraction peak. XRD patterns were obtained along the cutting speed (CD) direction, as well as the transversal one along the feed direction (TD). All measurements were carried out by the Proto iXRD Combo diffractometer (*Kα1* and *Kα2* of {211} planes, CrKα, Winholtz and Cohen method, ½s_2_ = 5.75 TPa^−1^, s_1_ = −1.25 TPa^−1^).

## 3. Results and Their Discussion

### 3.1. Cutting Force Components

**Figure 4:**
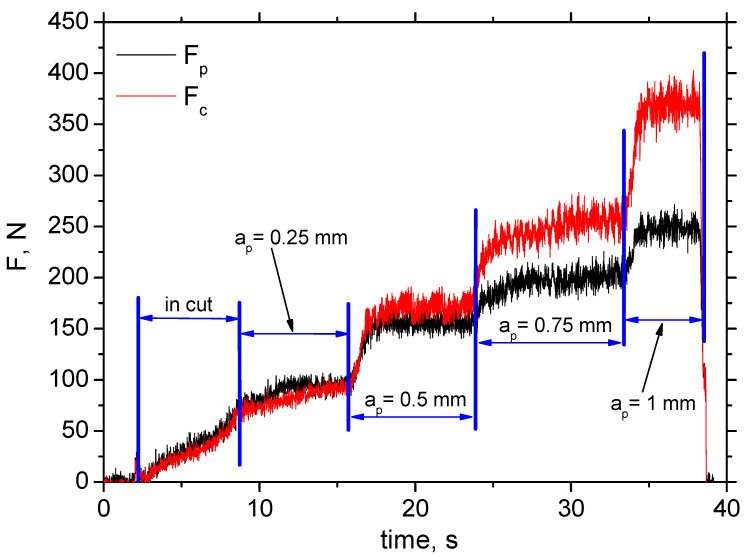
Filtered record of *F_c_* and *F_p_* as a function of cutting depth *a_p_*; insert P 15; *v_c_* = 50 m.min^−1^; *f* = 0.09 mm.

**Figure 5:**
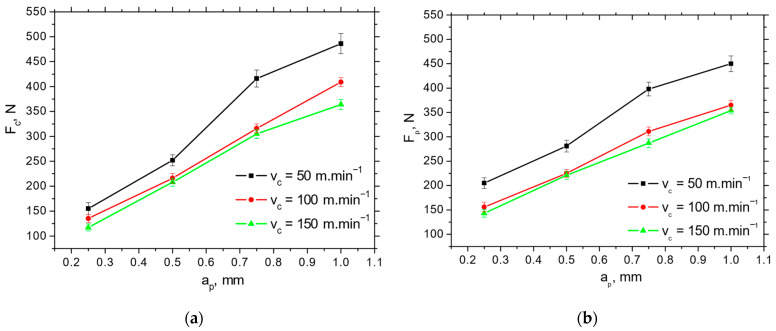
*F_c_* and *F_p_* extracted from the records, as depicted in Figure 4, as a function of the cutting speed *v_c_* and cutting depth *a_p_* for the insert IC 20; *f* = 0.09 mm. (**a**) *F_c_*; (**b**) *F_p_*.

**Figure 6:**
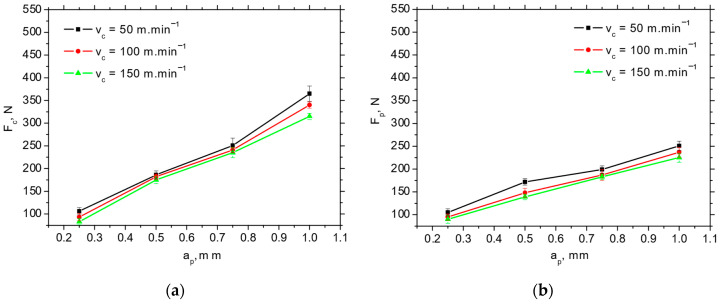
*F_c_* and *F_p_* extracted from records, as depicted in Figure 4, as a function of the cutting speed *v_c_* and cutting depth *a_p_* for insert P 15; *f* = 0.09 mm. (**a**) *F_c_*; (**b**) *F_p_*.

Figure 4 depicts the typical record of cutting force components *F_p_* and *F_c_* as a function of cutting depth, *a_p_*. Such records enable the extraction of these force components. The values of *F_p_* and *F_c_* are obtained at the end of each stage (bordered by blue lines) when the component values are stabilised. *F_p_* is less than *F_c_* at the higher *a_p_*, and both components drop down alongside cutting speed, *v_c_*, in the range from 19% (for the lower cutting depths) up to 23% (for the higher cutting depths) (see Figure 5 and Figure 6) due to the enhanced self-softening when the temperature in the cutting zone is increased with respect to the increasing heat flux. Such behaviour has been already proven by the measurements as reported earlier [15].

**Figure 7:**
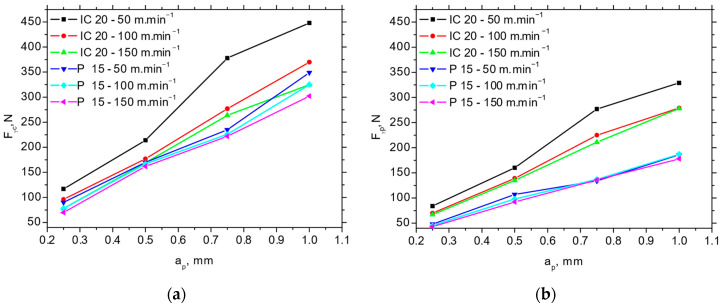
*F_γc_* and *F_γp_* as a function of cutting speed and cutting depth; *f* = 0.09 mm. (**a**) *F_γc_*; (**b**) *F_γp_*.

The *F_p_* and *F_c_* for IC 20 are more than those for P 15 (see Figure 5 and Figure 6), which in turn results in the lower *F_αtn_* and *F_αt_* (about 2.5 times higher for *F_αt_* and 1.8 times higher for *F_αtn_*) extracted from the obtained cutting force components using the aforementioned methodology depicted in Figure 1b (see Table 3). Furthermore, Figure 7 demonstrates that the components associated with chip formation and separation, *F_γc_* and *F_γp_*, are also lower for P 15 (about 30%) as compared with IC 20. The more significant drop in *F_p_* and *F_c_* for IC 20 when the cutting speed is increasing from 50 to 100 m.min^−1^ corresponds to the same behaviour with respect to *F_αtn_* and *F_αtn_*, as well as *F_γc_* and *F_γp_* (see Figure 5 and Table 3).

### 3.2. Chips Thickness and Shape

The diagrams of chip formation mainly show the function of feed *f* and cutting depth *a_p_*. Also, the aforementioned evolutions shown in Table 4 (in which information about *h_c_* as a function of cutting speed is provided) indicate that the influence of the cutting speed is quite limited. Chip formation strongly depends on the chip cross-sectional area, which is directly linked with the feed *f* and cutting depth *a_p_*. Taking into consideration the turning process kinematics, an investigation of the plastic deformation process in the cutting zone and the corresponding aspects should therefore be analysed as a function of the feed *f* and cutting depth *a_p_*.

**Figure 8:**
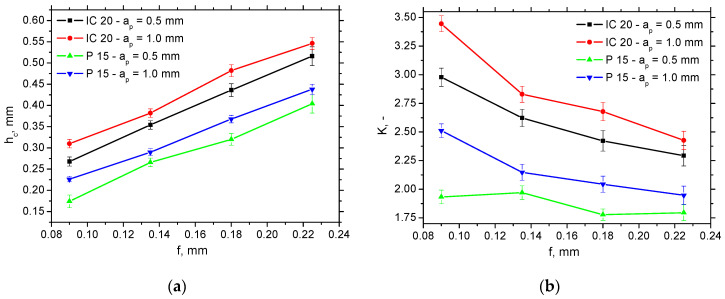
Chip thickness *h_c_* and chip ratio *K* versus feed and cutting depth for *v_c_* = 100 m.min^−1^. (**a**) *h_c_*; (**b**) *K*.

The thickness of the undeformed layer *h* (see Figure 1a) is equal to the feed *f*, and the chip thickness *h_c_* (see also Figure 1a) can be measured as mentioned earlier. Due to the variable chip thickness [16,17], the average values, such as those indicated in Figure 8a, were obtained from 20 repetitive measurements. This figure clearly indicates that the chip thickness *h_c_* for the insert IC 20 without a chip breaker is greater than that for P 15 (about 20%), and the influence of feed *f* is more than that of the cutting depth *a_p_*. Such behaviour results in the higher chip ratios, *K* (varies from 25% at the higher *f* up to 40% at the lower *f*) for the insert IC 20, as seen in Figure 8b, which can be calculated as follows:

(3) $$K=\frac{h_{c}}{h}\;\;\;\;\;\;\;(\text{-})$$

*K* is usually linked with the energy consumed for chip separation. For this reason, this process is more energy-demanding for the IC 20 insert as contrasted against the P 15 one. The differences among the *K* are statistically significant with respect to the indicated standard deviations presented (see Figure 8b). The statistical validity of the *K* parameter is directly linked with the validity of the data for chip thickness, *h_c_*, presented in Figure 8a, with respect to Equation (3).

Furthermore, the angle of primary plastic deformation, *Φ* (see Figure 1a), can be calculated as follows:

(4) $$\tan\Phi =\frac{\cos\gamma_{n}}{K-\sin\gamma_{n}}\;\;\;({}^{\circ})$$

where *γ_n_* is the effective value of *γ_n_* obtained from Table 2.

The chip speed *v_ch_* calculation is based on Equation (5), and the shear speed *v_sh_* on Equation (6).

(5) $$v_{ch}=v_{c}\cdot \frac{\sin\Phi }{\cos\left(\Phi -\gamma_{n}\right)}\;\;(m.\min^{-1})$$

(6) $$v_{sh}=v_{c}\cdot \frac{\cos\gamma_{n}}{\cos\left(\theta -\gamma_{n}\right)}\;\;(m.\min^{-1})$$

**Figure 9:**
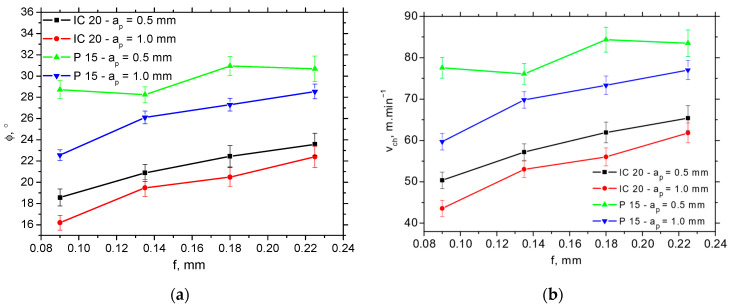
Angle of primary plastic deformation *Φ* and chip speed *v_ch_* as a function of feed and cutting depth for *v_c_* = 100 m.min^−1^. (**a**) *Φ*; (**b**) *v_ch_*.

**Figure 10:**
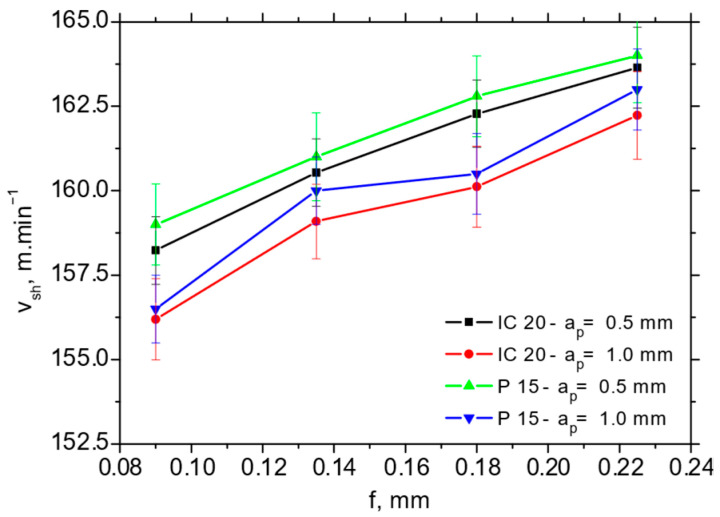
Shear speed *v_ch_* as a function of feed and cutting depth for *v_c_* = 100 m.min^−1^.

The higher *Φ* angles (such as those reported in Figure 9a) for the P15 insert take a strong role in the balance between the thermal and mechanical load, in which the chip is separated and formed afterwards. Figure 9b clearly demonstrates the higher chip speeds for the P15 insert (varies from 25% at the higher *f* up to 35% at the lower *f*), whereas the difference with respect to the shear speed (depicted in Figure 10) is only minor. Figure 10 demonstrates that the shear speed increases along the feed, but the differences with respect to the employed inserts as well as cutting depths are statistically insignificant.

**Figure 11:**
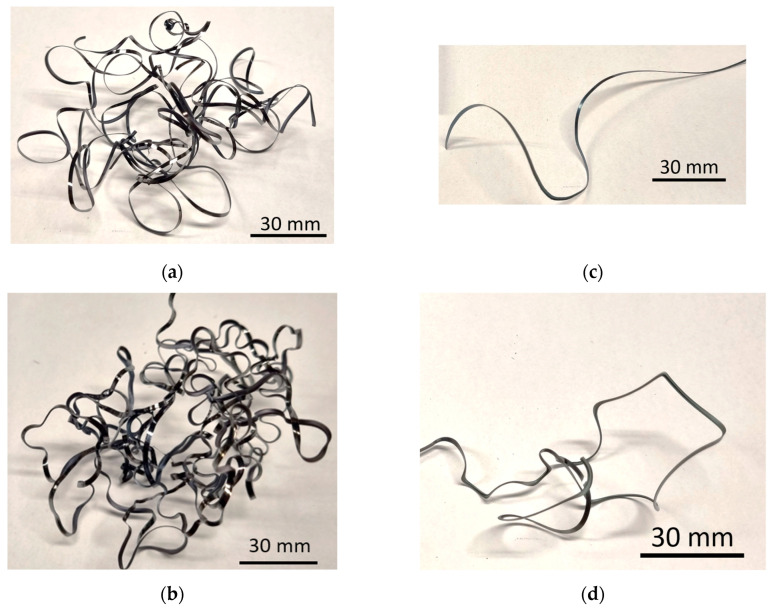
Chip shapes for *v_c_* = 100 m.min^−1^; *a_p_* = 1 mm. (**a**) P 15 and *f* = 0.09 mm; (**b**) P 15 and *f* = 0.225 mm; (**c**) IC 20 and *f* = 0.09 mm; (**d**) IC 20 and *f* = 0.225 mm.

Such evolution is closely related to the released heat, which is increasing along with the corresponding speed. It should be noted that plastic deformation under higher self-heating runs more easily due to the reduced hardness and strength of the machined material. For this reason, the chip thickness *h_c_* and the corresponding *K* are less for the insert P 15. On the other hand, this evolution is compensated by the energy consumed for chip formation for the insert P 15 due to the presence of the chip former. The well-shaped chips produced by the insert P 15 can be contrasted against the less curved ones for the insert IC 20 (see Figure 11). Less energy is consumed during the chip separation for the insert P 15, which is compensated by the less demanding process with respect to the chip formation for IC 20 and vice versa. However, the overall energy consumed for the chip formation and separation is more for the insert IC 20 due to the higher components *F_γc_* and *F_γp_*, resulting in the higher *F_γ_* (see Figure 7). Expressed in other words, two different aspects of the tool rake should be considered. The first one is linked with chip separation due to the dislocation shear along the angle *Φ*, and second one is linked with the energy consumed for chip shaping. Figure 11 clearly demonstrates that the energy necessary for the chip shaping in the case of the P 15 insert is more, as contrasted with the less curved chips produced by the IC 20 insert. On the other hand, the higher chip thickness *h_c_* for the IC 20 insert indicates that more energy is consumed for the chip separation with respect to the dislocation motion along *Φ*. The higher *F_γc_* and *F_γp_* for the insert IC 20 indicates that the influence of dislocation shear along *Φ* dominates, whereas the role of the energy consumed for chip shaping is only minor.

The influence of the cutting speed on *Φ* and *v_ch_* is directly linked with Equations (4) and (5). The reduced matrix strength at the elevated temperatures (at the higher *v_c_*) also reduces the energy consumed for plastic deformation in the cutting zone, which in turn decreases the *K* and makes the *Φ* higher. The increase in *v_c_* accelerated also *v_ch_* (see Equation (5)), while the decreasing *K* at the higher *v_c_* makes the chip thinner and longer.

### 3.3. Surface State

*F_αt_* and *F_αtn_*, indicated in Table 3, are directly related to the insert flank/machined surface interface. *F_αt_* is linked with the thermal load of the produced surface *Q_fl_* following Equation (7):

(7) $$Q_{fl}=\;F_{\alpha t}v_{c}\;\;\;\;(\mathrm{kJ}.s^{-1})$$

It should also be noted that the component linked with Equation (7) contributes to the overall heat developed in the cutting zone, supplemented by the heat developed due to dislocation motion along the angle *Φ* (usually the major heat source) and the heat developed due to the friction between the tool rake and the chip. It is well known that, during turning (at the cutting conditions listed in this manuscript), about 70% of the developed heat is dissipated by the chip, 25% by the machined materials, and about 4% by the tool (about 1% of the heat is irradiated into the environment in dry machining). The non-homogenous thermal field in the cutting zone and the heat partitioning are quasi-stationary. This means that the heat (and the corresponding temperature) field is stable within a certain time period. However, the heat distribution and the appearance of the thermal field can be valuably altered, especially due to the crater and flank wear due to the long- term interaction.

**Figure 12:**
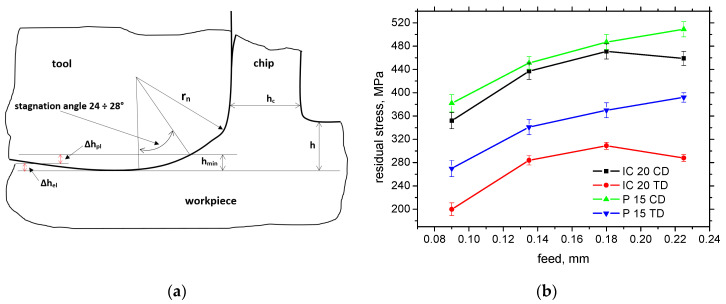
Illustration of *h_min_* and surface residual stress as a function of feed for *v_c_* = 100 m.min^−1^; *a_p_* = 1 mm. (**a**) Illustration of *h_min_*; (**b**) surface residual stress.

**Figure 13:**
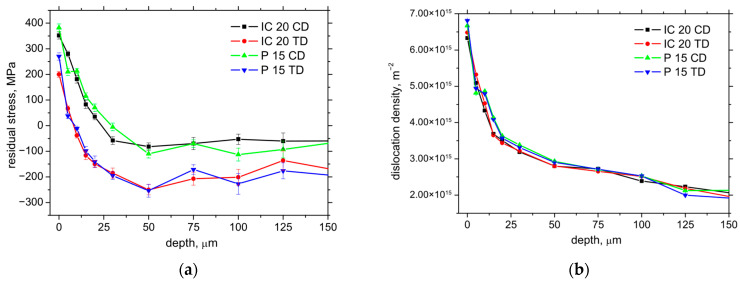
Depth profiles of residual stress as well as dislocation density; *v_c_* = 100 m.min^−1^; *a_p_* = 1 mm; *f* = 0.09 mm. (**a**) residual stress depth profile; (**b**) dislocation density depth profile.

On the other hand, the *F_αtn_* component represents the mechanical load of the produced surface. However, the cutting edge geometry (especially *r_n_*) is also closely related to the thickness of *h_min_*, as the layer undergoing the cutting edge is heavily deformed during the formation of the new surface (see Figure 12a). The final size of a produced component is given by the ratio between Δ*h_pl_* (the plastic component of *h_min_*) and Δ*h_el_* (the elastic component of *h_min_*); see Figure 12a. *h_min_* is mainly a function of *r_n_* when *h_min_* ≈ 0.26 *r_n_* [18,19]. Being so, the *h_min_* for IC 20 is about 2.3 μm, and it is 17.4 μm for P 15. Figure 12b depicts the lower surface tensile stresses for the IC 20 insert as a result of the higher *F_αtn_* component for this insert (about 10% lower for the CD and 25% for the TD). It should also be noted that the higher *F_αt_* and *F_αtn_* for IC 20 (despite the lower *r_n_*) are also due to the lower *γ_eff_* for this insert (see Table 2) and the qualitative transition in shear thermodynamics [20]. Furthermore, the presence of the TiN coating on the surface of the P 15 insert also valuably reduces the friction between the machined surface and the insert, as contrasted with the uncoated IC 20 (see Figure 2), which in turn contributes to the lower *F_αt_* and *F_αtn_* for the P 15 insert [21]. And, finally, it should also be noted that the extremely sharp cutting edge of the very low *r_n_* (in the case IC 20) is very unstable and rapidly rounded within quite a short time period. Such behaviour is associated with the very high contact stresses on the cutting edge, which dramatically drop down early during turning. This process strongly reduces the initial differences in *r_n_* and makes the real *r_n_* more comparable. On the other hand, these differences can be found mainly on the free surface, and the stress depth profiles are quite similar along CD and as TD; see Figure 13a.

XRD patterns can be used for the calculation of dislocation density *δ* as follows:

(8) $$\delta =\;\frac{1}{D^{2}}\;\;\;\;\;\;(m^{-2})$$

where *D* is the crystallite size obtained from Equation (9):

(9) $$D=\;\frac{K.\lambda }{\beta .\cos\theta }\;\;\;\;(\mathrm{nm})$$

where *K* is a constant equal to 0.89, *λ* is the X-ray wavelength, *β* is equal to the full width at half maximum of the XRD patterns, and *θ* is the Bragg angle.

The degree of strain accumulation expressed in terms of the dislocation density is nearly the same despite a significantly different *h_min_*, as reported earlier (see Figure 13b). Being so, the differences in the surface state expressed in terms of the residual stresses as well as the microstructure are mainly minor, and the different insert geometry mainly affects chip separation and formation. Quite a high magnitude of the tensile residual stresses (depicted on Figure 12b) on the free surface can have a negative influence on the fatigue strength during the real load of components [22]. On the other hand, this effect can be compensated by increasing the dislocation density (see also Figure 13b) [23]. It can also be noted that residual stress as well as strain accumulation are valuably altered during the subsequent heat and chemical treatment. The final surface state is mostly developed by the machining cycle after quenching and consequent tempering.

The elevated temperatures during turning (especially at the higher cutting speeds) might accelerate surface oxidation. The affected depth is usually within a few nanometres, and the XRD peaks linked with the presence of oxides are hidden in the background noise (cannot be recognised) of the irradiated region. For this reason, the presence of this thin layer on the free surface (containing oxides) has nearly no role with respect to the extracted residual stresses or the dislocation density.

It should also be noticed that the aforementioned behaviour with respect to the long-term interaction of the tools in the cutting zone might be affected by the progressive alterations in the insert shape and the corresponding geometry [1,9]. Crater wear on the tool rake develops a more positive geometry (rake angles), which in turn makes chip formation easier. Moreover, the accelerated self-heating also softens the machined matrix, which has a superimposing role in plastic deformation [7]. On the other hand, the progressive growth of the tool flank valuably increases the degree of plastic deformation in the machined surface. Such a process makes the dislocation density in the machined surface denser, and might initiate phase transformations and valuably alter the residual stress on the free surface and in the sub-surface layers [1,9,11]. The surface state expressed in the different terms is quite similar for both inserts due to the similar flank wear (*VB*)—below 0.05 mm. However, the valuable differences should be expected when the higher *VB* makes the mechanical and thermal load stronger, together with the time prolongation within the machined surface exposed to the thermo-mechanical load [1,9].

## 4. Conclusions

This study provides a deeper insight into chip separation and formation. It was found that the different insert geometries play quite a strong role in the interface between the insert rake and the chip. The study proves that the application of a suitable insert geometry valuably reduces the consumption of energy in the cutting zone, despite the fact that more energy is required to obtain the suitable chip form. This study can also be employed as a methodology for analyses of the different aspects in the cutting zone, focused on the investigation of surface integrity as well as plastic deformation in the cutting region. More valuable differences with respect to residual stresses or/and surface strain accumulation should be expected in cases of more developed tool flank wear, *VB*. This study investigates the interaction of produced surfaces and tool flanks mainly as a function of the cutting edge radius when the *VB* is very low, having a minor or nearly no role in the surface state. However, in real industrial practice, the progressive growth of *VB* should be expected, and the surface state, expressed in a variety of terms, would be altered. For this reason, the main focus of the incoming research should be related to the influence of *VB* on the surface state.

## Figures and Tables

**Table 1:** Chemical composition of the steel 16MnCr5 (wt. %).

Fe	C	Mn	Cr	S	P	Si	Cu
bal.	0.18	1.25	1	0.035	0.025	0.2	0.4

**Table 2:** Geometrical features extracted from the profiles, as depicted in Figure 1.

	IC 20	P 15
Mean radius of mean edge *r_n_*, mm	8.8	66.6
Clearance angle *α*, °	0 **(eff. 4)**	0.33 **(eff. 4.33)**
Wedge angle *β*, °	88.0	82.9
Rake angle *γ*, °	2.05 **(eff. −1,95)**	6.69 **(eff. 2.69)**

Note: **eff.** means the effective value due to the geometry of the tool holder −4°.

**Table 3:** *F_αt_* and *F_αtn_* as a function of cutting speed; *f* = 0.09 mm.

	IC 20			P 15		
*v_c_*	**50 m.min^−1^**	**100 m.min^−1^**	**150 m.min^−1^**	**50 m.min^−1^**	**100 m.min^−1^**	**150 m.min^−1^**
*F_αt_*	39 N	39 N	38 N	16 N	15 N	13 N
*F_αtn_*	121 N	86 N	76 N	65 N	50 N	47 N

**Table 4:** *h_c_* for IC20 as a function of feed and cutting depth for *a_p_* = 0.5 mm.

	*f* = 0.09 mm	*f* = 0.135 mm	*f* = 0.180 mm	*f* = 0.225 mm
*v_c_* = 50 m.min^−1^	0.29 ± 0.009 mm	0.38 ± 0.011 mm	0.46 ± 0.016 mm	0.54 ± 0.023 mm
*v_c_* = 100 m.min^−1^	0.27 ± 0.010 mm	0.35 ± 0.010 mm	0.43 ± 0.015 mm	0.51 ± 0.022 mm
*v_c_* = 150 m.min^−1^	0.26 ± 0.008 mm	0.33 ± 0.007 mm	0.41 ± 0.012 mm	0.49 ± 0.018 mm

## Data Availability

The data available on the special request.
